# Distinction between clonal and paraclonal cutaneous involvements in VEXAS syndrome

**DOI:** 10.1186/s40164-022-00262-5

**Published:** 2022-02-16

**Authors:** Valentin Lacombe, Annaelle Beucher, Geoffrey Urbanski, Yannick Le Corre, Laurane Cottin, Anne Croué, Anne Bouvier

**Affiliations:** 1 Service de Médecine Interne et Immunologie Clinique, Centre Hospitalier Universitaire, 4 rue Larrey, Angers, France; 2 Mitolab Team—CNRS 6214—INSERM 1083—MITOVASC Institute, University of Angers, Angers, France; 3 Laboratoire d’Hématologie, Centre Hospitalier Universitaire, Angers, France; 4 Fédération Hospitalo-Universitaire ‘Grand Ouest Against Leukemia’ (FHU GOAL), Angers, France; 5 Service de Dermatologie, Centre Hospitalier Universitaire, Angers, France; 6 CRCINA, INSERM, SFR ICAT, Universités d’Angers, Angers, France; 7 Département de Pathologie Cellulaire et Tissulaire, Centre Hospitalier Universitaire, Angers, France

**Keywords:** Autoinflammatory diseases, Sweet syndrome, Vasculitis, Clonal hematopoiesis, Mutation

## Abstract

VEXAS (vacuoles, E1 enzyme, X-linked, auto-inflammatory, somatic) syndrome is an inflammatory disorder with hematological and systemic features. A recent study demonstrated that the dermal infiltrate in neutrophilic dermatosis from VEXAS patients is derived from the pathological *UBA1*-mutated myeloid clone. Neutrophilic dermatosis is, however, only one of the various skin involvements observed in VEXAS syndrome. We analyzed 10 formalin-fixed paraffin-embedded skin biopsies from genetically confirmed VEXAS syndrome. *UBA1* mutation was found in the biopsies related to neutrophilic dermatitis but in none of the other histological patterns (leukocytoclastic vasculitis and septal panniculitis). This could lead to a distinction between clonal and paraclonal cutaneous involvements in VEXAS syndrome, which could in turn improve therapeutic outcomes.

## To the Editor,

VEXAS (vacuoles, E1 enzyme, X-linked, auto-inflammatory, somatic) syndrome is a recently described adult-onset inflammatory disorder with various involvements including arthritis, chondritis, cutaneous features, macrocytic anemia and myelodysplastic syndrome [1–4]. Zakine et al. recently performed molecular analyses on 8 paraffin-embedded skin tissue sections of neutrophilic dermatosis in patients with VEXAS syndrome [5]*.* They identified *UBA1* mutations in all of these skin samples. This was the first study to demonstrate a strong link between the presence of *UBA1*-mutated cells in an involved tissue (except for bone marrow) and the related clinical manifestations. According to the author’s conclusion, this suggests that the dermal infiltrate in VEXAS skin lesions is derived from the pathological myeloid clone, which could be targeted to treat VEXAS patients with cutaneous involvement.

Neutrophilic dermatosis is, however, only one of the various skin involvements observed in VEXAS syndrome. Indeed, leukocytoclastic vasculitis, erythema nodosa and periorbital edema have also been reported in this rare and recently described disorder [1]. Consequently, we aimed to assess the presence and abundance of the *UBA1*-mutated clone in the different cutaneous involvements related to VEXAS syndrome.

We retrospectively analyzed the medical record of 6 patients with both genetically confirmed VEXAS syndrome (*UBA1* mutation identified from blood samples, *UBA1* variants detailed in Table 1) and related skin involvement with formalin-fixed paraffin-embedded skin biopsies performed between January 2017 and September 2021 in Angers University Hospital. Two methods of dewaxing and DNA extraction were used (Kit NucleoSpin Tissue and NucleoSpin DNA FFPE XS, Macherey Nagel, Düren, Germany). The quality and quantity of the extracted DNA samples were evaluated using a NanoDrop 1000 spectrophotometer (Thermo Fisher Scientific, Waltham, USA). In case of sufficient DNA quantity and quality (260/280 nm absorbance ratio between 1.8 and 2.0), somatic mutations in *UBA1* (NM_003334.3) [1, 6] were then screened by Sanger sequencing (BigDye^TM^ Terminator v3.1 Cycle Sequencing Kit with 3130xl genetic analyzer, Applied Biosystems^TM^, USA), as previously described [7]. The minimal variant allele frequency (VAF) allowing *UBA1* mutations to be detected with Sanger sequencing was determined to be 10% by diluting *UBA1*-mutated DNA samples with known VAF assessed by Next Generation Sequencing.

**Table 1:** Clinical and histological features of included patients and biopsies, and results of the DNA extraction and sequencing

Skin biopsy specimen number	Treatment at the time of the biopsy	Clinical features	Histological pattern	DNA extraction of quality?	Sanger sequencing results
Patient #1 (male, 78 years-old, *UBA1* mutation p.Met41Thr)					
No. 1	None	Erythema nodosum	Septal panniculitis	Yes	*UBA1*-wild type
No. 2	None	Erythema nodosum	Septal panniculitis	Yes	*UBA1*-wild type
No. 3	Cortancyl	Papules	Neutrophilic dermatosis	Yes	*UBA1*-mutated
Patient #2 (male, 72 years-old, *UBA1* mutation p.Met41Thr)					
No. 1	None	Papules	Leukocytoclastic vasculitis	No	NA
Patient #3 (male, 63 years-old, *UBA1* mutation p.Met41Leu)					
No. 1	Methotrexate	Papules, nodules	Leukocytoclastic vasculitis	Yes	*UBA1*-wild type
No. 2	Methotrexate, abatacept	Papules, purpura	Leukocytoclastic necrotizing vasculitis	Yes	*UBA1*-wild type
No. 3	Etanercept	Papules	Neutrophilic dermatosis	Yes	*UBA1*-mutated
Patient #4 (male, 64 years-old, *UBA1* mutation p.Met41Val)					
No. 1	None	Erythema nodosum	Septal panniculitis	No	NA
No. 2	None	Livedo	Subnormal	No	NA
Patient #5 (male, 87 years-old, *UBA1* mutation p.Met41Val)					
No. 1	None	Macules, purpura	Leukocytoclastic vasculitis	No	NA

**Figure 1:**
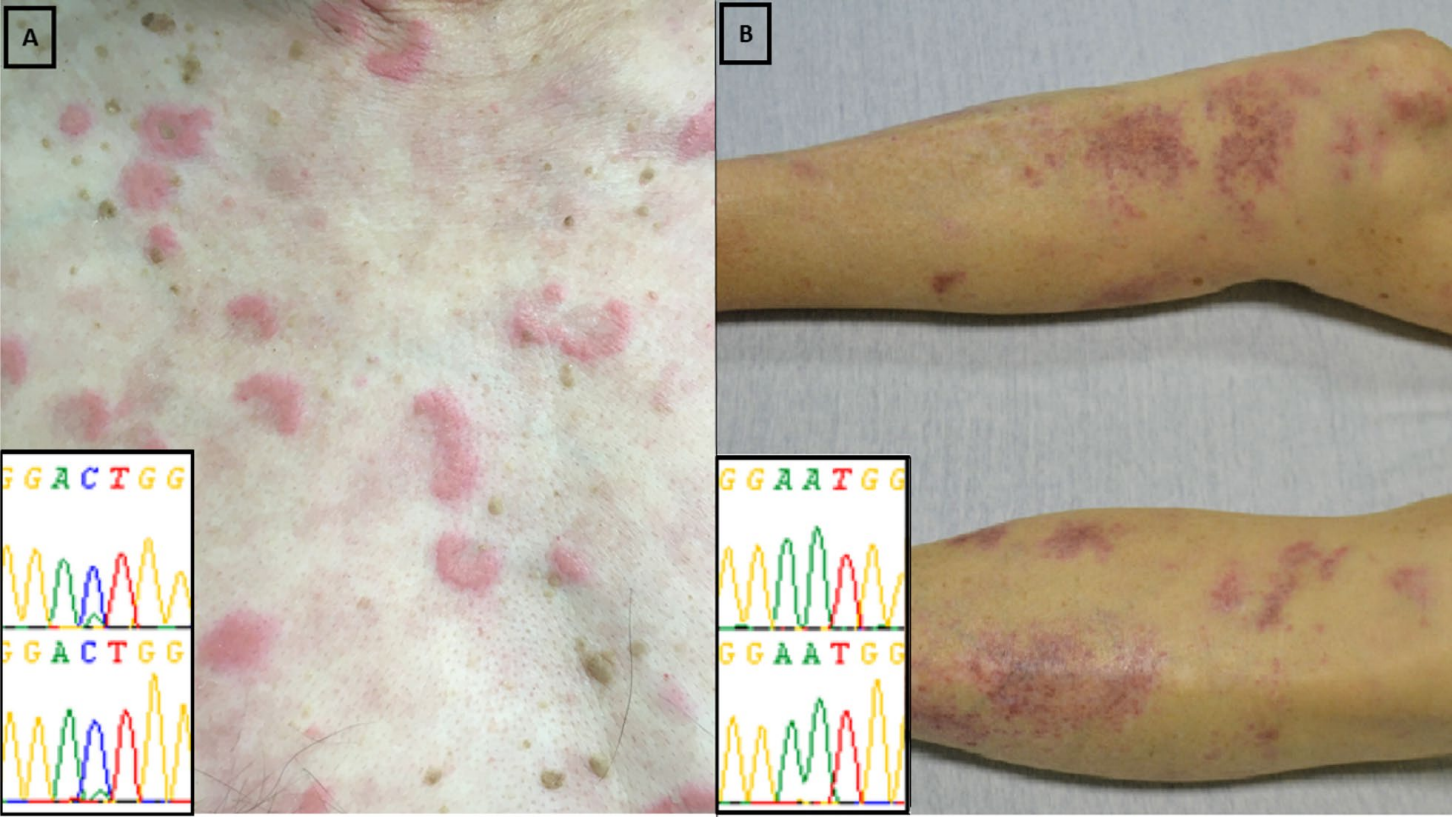
Results of molecular analysis with Sanger sequencing according to the type of skin involvement. **A** presents the *UBA1* mutation (p.Met41Leu, c.121A>C, mutational load >50%) observed with Sanger sequencing from a skin biopsy in a patient with VEXAS syndrome and neutrophilic dermatosis. **B** presents the *UBA1*-wild type gene observed in a skin biopsy in a patient with VEXAS syndrome and leukocytoclastic vasculitis

Ten skin biopsies were performed in the period of interest and involved either erythema nodosum, papules, nodules, purpuric macules or livedo. The clinical, molecular and pathological pattern of the included patients and biopsies were detailed in Table 1. The DNA extraction allowed sequencing in 6/10 samples from biopsies with the following histological patterns: 2 neutrophilic dermatosis (intense and diffuse neutrophilic infiltrate with no evidence of infection or vasculitis), 2 leukocytoclastic vasculitis (angiocentric segmental inflammation with fibrinoid necrosis and neutrophilic infiltrate with karyorrhexis in the small vessel walls), and 2 septal panniculitis (inflammatory cell infiltrate at the periphery of the hypodermal lobules). *UBA1* mutation was found in the 2 biopsies related to neutrophilic dermatitis but in none of the other histological patterns (Figure 1). The mutational load was higher in neutrophilic dermatosis (> 50%) than in blood or bone marrow samples (< 50%) from the same patient. Associated hematological features were as follows: anemia (6/6), macrocytosis (4/6), thrombocytopenia (3/6), neutropenia (3/6), lymphopenia (3/6) and myeodysplastic syndrome (2/6, patients #1 and #4).

Previous studies specified the clinical contexts that should lead to searching for a *UBA1* mutation in blood or marrow samples to confirm suspected VEXAS syndrome [4, 8], and demonstrated that the *UBA1* mutated myeloid clone may also infiltrate the VEXAS-related skin lesions [5]. In our pilot study, we confirmed the presence of *UBA1*-mutated cells in skin tissues in cases of neutrophilic dermatosis. However, we did not identify any *UBA1* variation in the four other biopsies with different histological patterns despite a sensitivity to identify the mutation in case of > 10% mutated cells. While we cannot rule out the idea that the absence of UBA1 mutation was related to a lower myeloid infiltrate in non-neutrophilic dermatosis lesions, we demonstrated that the *UBA1*-mutated clone is either absent or much less abundant in non-neutrophilic dermatosis skin lesions. We could hypothesize the distinction between “clonal” (neutrophilic dermatosis) and “paraclonal” (leukocytoclastic vasculitis and septal panniculitis) cutaneous involvements in VEXAS syndrome. This could result in different treatment options using clonal-depleting therapy in neutrophilic dermatosis, whereas paraclonal involvements could be treated with drugs targeting the inflammation and cytokine release. Indeed, Commont et al. recently showed promising results about the efficiency of azacitidine for treating patients with VEXAS syndrome [9]. This type of treatment could be particularly useful in patients with clonal involvements.

In conclusion, the *UBA1*-mutated clone was observed in VEXAS-related skin involvement in cases of neutrophilic dermatosis but not in other histological patterns. This could lead to a distinction between clonal and paraclonal cutaneous involvements in VEXAS syndrome, with which could in turn improve therapeutic outcomes.

## Data Availability

The datasets used and/or analyzed during the current study are available from the corresponding author on reasonable request.

## Abbreviation

VAF: variant allele frequency
